# Immunomodulatory Effects of the Tobacco Defensin NaD1

**DOI:** 10.3390/antibiotics13111101

**Published:** 2024-11-19

**Authors:** Ekaterina I. Finkina, Ivan V. Bogdanov, Olga V. Shevchenko, Serafima I. Fateeva, Anastasia A. Ignatova, Sergey V. Balandin, Tatiana V. Ovchinnikova

**Affiliations:** 1 M.M. Shemyakin and Yu.A. Ovchinnikov Institute of Bioorganic Chemistry, Russian Academy of Sciences, 117997 Moscow, Russiaovch@ibch.ru (T.V.O.); 2 Moscow Center for Advanced Studies, 123592 Moscow, Russia; 3 Department of Bioorganic Chemistry, Lomonosov Moscow State University, 119991 Moscow, Russia

**Keywords:** antimicrobial peptides (AMPs), host defense peptides, plant defensins, tobacco defensin NaD1, pea defensin Psd1, human β-defensin 2 (HBD2), human cathelicidin LL-37, immunomodulatory effects, lipopolysaccharide (LPS), zymosan, cytokines

## Abstract

**Background/Objectives:** Defensins are important components of the innate plant immune system, exhibiting antimicrobial activity against phytopathogens, as well as against fungi pathogenic to humans. Along with antifungal activity, plant defensins are also capable of influencing various immune processes, but not much is known about these effects. In this study, we investigated the immunomodulatory effects of the tobacco defensin NaD1, which possesses a pronounced antifungal activity. **Methods and Results:** We showed that NaD1 could penetrate the Caco-2 polarized monolayer. Using a multiplex assay with a panel of 48 cytokines, chemokines and growth factors, we demonstrated that NaD1 at a concentration of 2 μM had immunomodulatory effects on human dendritic cells and blood monocytes, mainly inhibiting the production of various immune factors. Using the sandwich ELISA method, we demonstrated that NaD1 at the same concentration had a pronounced immunomodulatory effect on unstimulated THP-1-derived macrophages and those stimulated by bacterial LPS or fungal zymosan. NaD1 had a dual effect and induced the production of both pro-inflammatory cytokine IL-1β as well as anti-inflammatory IL-10 on resting and pro-inflammatory THP-1-derived macrophages. We also found that the immunomodulatory effects of the tobacco defensin NaD1 and the pea defensin Psd1 differed from each other, indicating nonuniformity in the modes of action of plant defensins. **Conclusions:** Thus, our data demonstrated that the tobacco defensin NaD1 exhibits different immunomodulatory effects on various immune cells. We hypothesized that influence on human immune system along with antifungal activity, could determine the effectiveness of this peptide under infection in vivo.

## 1. Introduction

It is well known that host defense antimicrobial peptides (AMPs) such as human defensins and cathelicidins not only effectively inhibit the growth of pathogenic microorganisms but also possess immunomodulatory activity, which helps to prevent infection. In addition, it has been shown that a number of AMPs from marine organisms [1], insects [2], plants [3] and others also exhibit immunomodulatory properties. Recent data demonstrated that plant defensins were also able to influence the human immune system [4].

Plant defensins are AMPs which protect plants against pathogen and parasite invasion. Plant defensins effectively inhibit the growth of human pathogenic fungi of the *Candida*, *Aspergillus* and *Cryptococcus* genera, which, according to phenotypic tests, PCR assays and metagenomic-based studies, are a common cause of life-threatening fungal infections [5]. Nowadays, plant defensins are considered as high-potential prototypes of new antifungal drugs [4,6]. These AMPs have similar spatial organizations but are characterized by low amino acid sequence homology, which is probably the reason for the diversity of mechanisms of their antifungal action [4].

Several plant defensins have been demonstrated to have immunomodulatory action on epithelial, immune and endothelial cells. γ-Thionin from *Capsicum chinense* upregulated the expression of TLR2 receptor, cytokines TNF-α, IL-1β and IL-10 and also activated the transcriptional factors of inflammatory response in untreated bovine mammary epithelial cells (bMECs) and those infected by *Staphylococcus aureus* [7,8]. This peptide significantly reduced the internalization of *S. aureus* cells into bMECs, which was not related to its antibacterial activity [7]. The peptide solyC, based on the γ-motif of tomato defensins, exhibited an anti-inflammatory activity, decreasing the production of pro-inflammatory cytokines by LPS-stimulated THP-1 cells [9]. The pea defensin Psd1 up-regulated the expression of human β-defensin 2 (HBD-2) and pro-inflammatory cytokines in epithelial cells Caco-2, decreasing the effects of *Candida albicans* [10]. The pea Psd1 and the lentil Lc-def defensins induced the production of both pro- and anti-inflammatory cytokines/chemokines and growth factors by monocyte-derived dendritic cells and monocytes [10,11]. PaDef from avocado and γ-thionin from *C. chinense* reduced the VEGF-induced proliferation of bovine endothelial cells, as well as other effects of pro-angiogenic factor VEGF [12].

Floral defensin NaD1, a peptide with pronounced antifungal activity, is found in tobacco, which is known to be abundantly rich in biologically active phytochemicals [13]. This peptide is characterized by a complex mechanism of antifungal action, interacts with the fungal cell wall, affects the permeability of the plasma cell membrane by interacting with phosphatidylinositol-4,5-bisphosphate (PI(4,5)P2), penetrates into the cell and causes oxidative cell stress [14,15]. As shown, the development of yeast resistance to NaD1 can take place, but it arises more slowly than to the conventional antimycotic caspofungin [16]. At the same time, to date, nothing is known about the immunomodulatory effects of the tobacco defensin NaD1.

The main goal of this study was to investigate the immunomodulatory action of NaD1. At the first stage, we investigated the ability of NaD1 to penetrate the epithelial barrier by using a monolayer Caco-2 cells model. The effects of this peptide on interleukin production by such immune cells as monocytes and dendritic cells were studied by using the multiplex xMAP assay. Finally, the effects of NaD1 on THP-1-derived macrophages under inflammation caused by such pathogen-associated molecular patterns (PAMPs) as the lipopolysaccharide (LPS) of Gram-negative bacteria and zymosan from yeast cell walls were studied by using the sandwich ELISA method. The host defense antimicrobial peptides HBD2 and LL-37, produced by epithelial and immune cells, as well as the pea defensin Psd1, exhibiting immunomodulatory activity, were used by way of comparison.

## 2. Results and Discussion

### 2.1. Cytotoxic Effects of NaD1 Towards Epithelial and Immune Cells

It was shown previously that the tobacco defensin NaD1 exhibited cytotoxic properties. The IC_50_ for umbilical vein endothelial cells (HUVEC), human smooth muscles (CASMC) and human dermal fibroblast cells (AHDF) was approximately 10 µM in the MTT cell viability assay [17]. Therefore, we investigated the cytotoxicity of NaD1 towards epithelial and immune cells by using the resazurin-based method to exclude cytotoxic effects of the peptide on these cells at concentrations of 0.2, 2 and 5 µM applied in subsequent experiments. The membrane-active peptide melittin from the venom of honeybees, exhibiting high hemolytic and nonspecific cytotoxic activity [18], was used for comparation.

**Figure 1:**
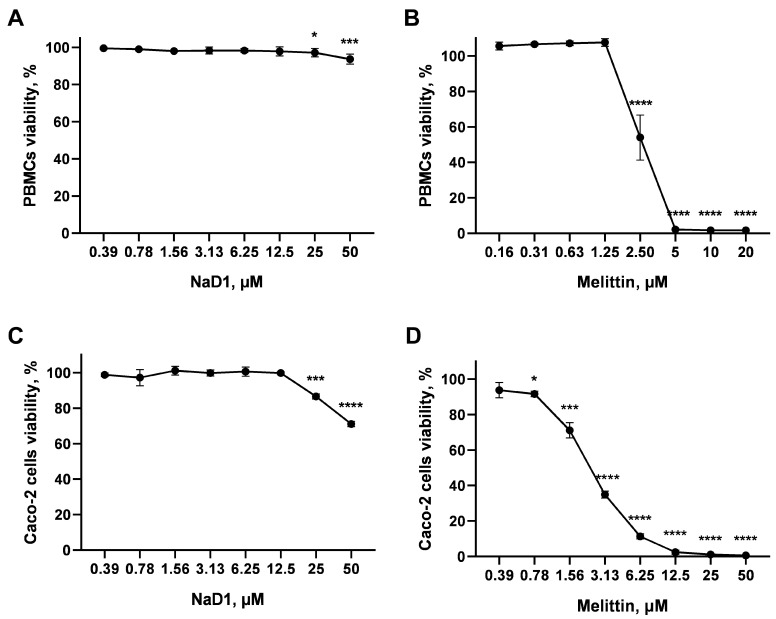
Cytotoxic effects of the tobacco defensin NaD1 towards PBMCs (**A**) and Caco-2 cells in monolayer (**C**). The membrane-active peptide melittin from the venom of honeybees (**B**,**D**) was used for comparison. Error bars represent a standard deviation (±SD) between two biological and two technical replications. Significance levels are * *p* ≤ 0.05, *** *p* < 0.001 and **** *p* < 0.0001. The significance was calculated by comparing untreated cells (control) with treated by NaD1 or melittin cells. Viability cells in control and experimental samples was compared with un-paired two-sample *t*-test.

Human peripheral blood mononuclear cells (PBMCs) were chosen as a commonly used heterogeneous population of immune cells, consisting of lymphocytes (B cells, T cells and NK cells) and a smaller fraction of monocytic and dendritic cells. NaD1 had a rather low cytotoxic activity on PBMCs. Cell viability of approximately 90% was observed at the peptide concentration of 50 µM (Figure 1A). Even at NaD1 concentration of 180 μM, the viability of PBMCs was more than 50% (Supplementary Materials, Figure S1A). At the same time, melittin induced approximately 50% cell death at a concentration of 2.5 µM (the calculated cytotoxic concentration of melittin corresponding to 50% viability of PBMCs (CC_50_) was 2.54 µM) (Figure 1B).

A Caco-2 monolayer mimicking the gastrointestinal epithelial barrier was also used in the cytotoxic assay. The tobacco defensin NaD1 exhibited cytotoxic effects towards the Caco-2 monolayer, but at rather high concentrations; it had no effects on the Caco-2 cells in a monolayer at a concentration of 12.5 µM. Cell viability of approximately 85% and 70% was observed at the peptide concentrations of 25 and 50 µM, respectively (Figure 1C). For comparison, a decrease in cell viability was observed even at a concentration of melittin of 0.8 µM. Cell viability was less than 40% at a concentration of this peptide of 3.1 μM (the calculated CC_50_ of melittin was 2.4 µM) (Figure 1D). A similar situation was observed in the case of Caco-2 cells not in a monolayer. Cell viability of approximately 62% was observed at a NaD1 concentration of 53.3 µM. A significant increase in cell death was observed at high peptide concentrations. Cell viability of only 8% was observed at a NaD1 concentration of 180 μM (the calculated CC_50_ of NaD1 was 61.8 µM) (Supplementary Materials, Figure S1B).

Thus, NaD1 did not exhibit a cytotoxic effect against the tested immune and epithelial cells at a concentration of 12.5 µM. Earlier, it had been shown that the human defensin HBD2 did not increase the level of THP-1 cell death at the concentration of 1.2 µM [19]. The human cathelicidin LL-37 has been previously shown to have a toxic effect on PBMCs at the concentration of 12 µM [20]. The pea defensin Psd1, as we have shown in a previous study, had no cytotoxic activity against PBMCs at the concentration of 50 µM [10].

### 2.2. Transfer of the Tobacco Defensin NaD1 Across the Caco-2 Polarized Monolayer

**Figure 2:**
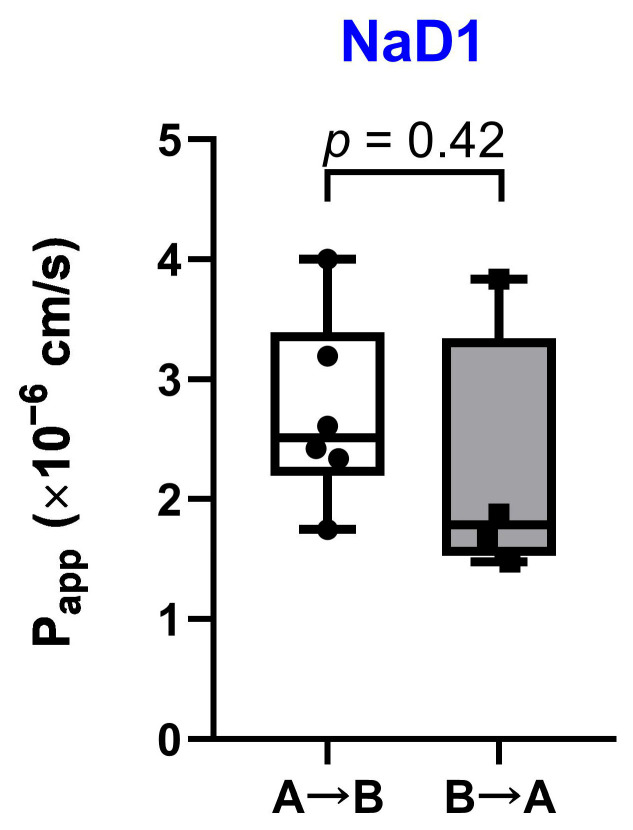
Assessment of bidirectional transport of the tobacco defensin NaD1 through the polarized Caco-2 monolayer. A→B, absorptive transport; B→A, secretory transport; Papp—apparent permeability coefficient. Six and four independent biological replications were used for absorptive and secretory directions, respectively. The normality of Papp coefficient distribution was assessed using Shapiro–Wilk test. Papp coefficients were compared by unpaired two-sample *t*-test.

To find out whether the tobacco defensin NaD1 can penetrate epithelial barriers we evaluated the permeability of the Caco-2 polarized monolayer to this peptide. Bidirectional transport of NaD1 at the concentration of 5 μM through the polarized Caco-2 monolayer was assessed in two directions: (1) from the apical to basolateral chamber (A→B, absorptive) and (2) from the basolateral to apical chamber (B→A, secretory) (Figure 2).

Mean values of apparent permeability coefficients for absorptive and secretory directions were quite similar: 2.72 × 10^−6^ and 2.22 × 10^−6^ cm/s, respectively. Almost equal permeability in both directions suggests the passive transport of NaD1 through the Caco-2 monolayer. As we have shown earlier, the pea defensin Psd1 is probably subjected to active efflux, as apparent permeability coefficients were higher in the secretory direction than in the absorptive one [10]. This may mean that Psd1 is probably a weak substrate of some efflux pumps in Caco-2 cells. At the same time, the apparent permeability coefficients of NaD1 and Psd1 for A→B direction were nearly the same. This difference in the transfer of NaD1 and Psd1 through the Caco-2 polarized monolayer could be explained by discrepancies in their structural organization. It is well known that despite the similarity in spatial structures, plant defensins do not contain conserved regions in their amino acid sequences (Supplementary Materials, Figure S2) [4]. In particular, the primary structure of the tobacco NaD1 is very different from that of the pea Psd1 and has only 26% homology with the latter. In addition, the Psd1 isoelectric point is 7.73, in contrast to that of the more cationic NaD1 (pI 9.08).

Thus, based on the data obtained, we assumed that NaD1 is able to transfer across the human intestinal epithelium and interact with immune cells.

### 2.3. Immunomodulatory Action of the Tobacco Defensin NaD1 on Human Dendritic Cells and Blood Monocytes

**Figure 3:**
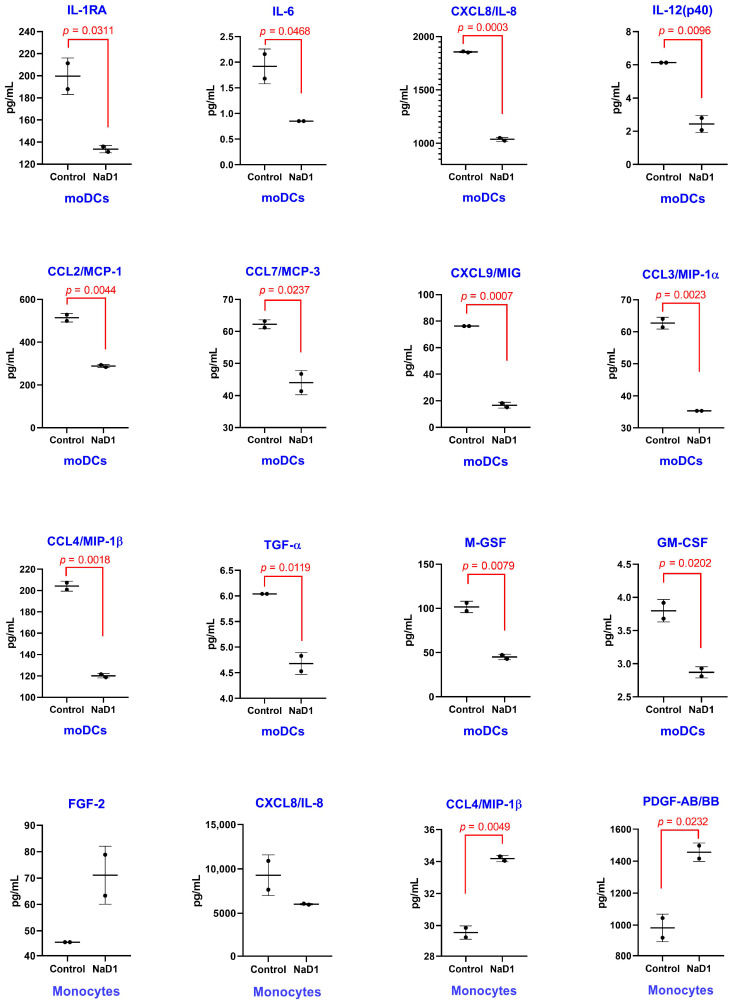
Production of cytokines, chemokines and growth factors upon stimulation of DCs and monocytes by NaD1 at the concentration of 2 μM. Error bars represent a standard deviation (±SD) between two biological replications. The levels in control and experimental wells were compared by unpaired two-sample *t*-test.

In the next step of this work, we studied the immunomodulatory properties of NaD1 by using primary monocytes and monocyte-derived immature dendritic cells (DCs). DCs represent a type of antigen-presenting cells, playing a key role in adaptive immune response. Their primary functions involve phagocytosis, processing and presentation of captured antigens to T cells. In most tissues, they are present in a state known as “immature” DCs, unable to activate and stimulate T lymphocytes; their activation and maturation typically starts when DCs identify danger signals [21]. This is why immature DCs are present where antigen entrance is expected; for instance, in lower and upper airways, skin and gut epithelial barriers, etc. In the case of immature DCs, NaD1 at the concentration of 2 μM mainly inhibited the production of cytokines and chemokines, including ones with pro- and anti-inflammatory action: IL-1RA (from 199 to 133 pg/mL, *p* = 0.0311), CXCL8 (from 1856 to 1037 pg/mL, *p* = 0.0003), CCL2 (from 514 to 288 pg/mL, *p* = 0.0044), CCL7 (from 62.22 to 44.12 pg/mL, *p* = 0.0237), CXCL9 (from 76.28 to 16.72 pg/mL, *p* = 0.0007), CCL3 (from 62.72 to 35.36 pg/mL, *p* = 0.0023), CCL4 (from 204 to 120 pg/mL, *p* = 0.0018) and M-CSF (from 101 to 45.07 pg/mL, *p* = 0.0079). In the case of IL-6, IL-12(p40), TGF-α and GM-CSF, a very slight but statistically significant inhibition of the production was observed (Figure 3). Apparently, NaD1 did not induce the activation and maturation of immature DCs, according to their cytokine profiles. In our previous studies of the immunomodulatory properties of plant defensins, we have shown that the pea defensin Psd1, on the contrary, induced an elevation of the production of cytokines IL-1RA, IL-5, IL-6, IL-10, IL-12, IL-15, IL-27 and TNFα and chemokines CXCL8/IL-8, CCL2/MCP-1 and CCL7/MCP-3 by mature DCs [9]. At the same time, the lentil defensin Lc-def, having 48% and 34% homology with the pea Psd1 and the tobacco NaD1, respectively, did not induce significant changes in the secretion of cytokines and chemokines by mature DCs [11].

Then, primary human monocytes were chosen to study the immunomodulatory properties of NaD1. Monocytes circulating in the blood can be recruited and extravasated into tissues during inflammatory processes [22]. Moreover, the recruitment of monocytes to the sites of inflammation is critical for host defense not only when infected, but also in the case of sterile injury [22]. In the case of monocytes, NaD1 at the same concentration induced a decrease of chemotactic CXCL1/GROα and CXCL8/IL-8 levels and elevation of the fibroblast growth factor 2 (FGF-2) production; however, these changes were not statistically significant (Figure 3). At the same time, NaD1 induced a slight but statistically significant (*p* = 0.0049) elevation of the production of pro-inflammatory CCL4/MIP-1β (from 29.57 to 34.19 pg/mL), which is a potent monocyte and lymphocyte chemoattractant, recruiting neutrophils, eosinophils, basophils, immature dendritic cells and natural killer cells to the site of inflammation [23]. NaD1 also increased the production of the platelet-derived growth factor (PDGF-AB/BB) (from 984 to 1457 pg/mL, *p* = 0.0232), which is a potent mitogen for cells of mesenchymal origin and plays a significant role in angiogenesis and wound healing [24]. NaD1 also induced an elevation of the PDGF-AA level, but this effect was not statistically significant (Supplementary Materials, Table S1). Interestingly, the lentil defensin Lc-def also increased the production of PDGF-AA, PDGF-AB/BB and CCL3/MIP-1α by monocytes [11], while the pea defensin Psd1 increased the production of both CCL3/MIP-1α and CCL4/MIP-1β by monocytes [10].

According to the cytokine profile, NaD1 appeared to be not capable of activating and inducing the maturation of dendritic cells. Instead, NaD1 inhibited the production of cytokines by immature DCs, and this effect was statistically significant. In primary monocytes, NaD1 also induced minor effects. Other plant defensins, such as the pea Psd1 and the lentil Lc-def, induced much more significant changes in cytokine production by mature DCs and monocytes, suggesting that the tobacco NaD1 causes mild immunomodulatory effects on these cell cultures. Taking these results together, we concluded that representatives of plant defensins could induce diverse immunomodulatory effects on various human immunocompetent cells.

### 2.4. Immunomodulatory Action of the Tobacco Defensin NaD1 on Unstimulated and Stimulated by LPS or Zymosan THP-1-Derived Macrophages

Next, we investigated the effects of NaD1 on THP-1-derived macrophages under inflammation caused by bacterial or fungal PAMPs, such as LPS and zymosan. LPS is the most abundant component of the cell wall of Gram-negative bacteria and an agonist of the TLR4 receptor, which stimulates the production of TNF-α, IL-1β, IL-6, IL-8 and other inflammatory cytokines in various cell types in response to pathogens [25]. Zymosan prepared from yeast cell walls mainly consists of polysaccharides including β-glucan and, to a lesser extent, of mannan and activates secretion of inflammatory factors, in particular TNF-α and IL-8, by macrophages, monocytes and leukocytes via TLR2 and Dectin-1 receptors [26]. Macrophages maintain tissue homeostasis, resist pathogen invasion and, therefore, are one of the key players in tissue immunity [27]. They are present in different tissues and can be activated and polarized depending on their environment into pro-inflammatory (M1) or anti-inflammatory macrophages (M2) [28]. LPS or pro-inflammatory cytokines (IFNγ, IL-12) induce the polarization of macrophages with the resting phenotype (M0) to the M1 phenotype while anti-inflammatory cytokines (IL-4, IL-10 and IL-13) can drive their polarization towards M2 [27]. Recently, it has been shown that zymosan induced upregulation of pro-inflammatory genes, intrinsic to M1 macrophages, and downregulated the M2 genes [29]. Host defense cationic antimicrobial peptides with immunomodulatory properties, which are produced by epithelial and immune cells, namely the human β-defensin HBD2 and the cathelicidin LL-37, were used for comparison. The pea defensin Psd1 was also used in this experiment. Previously, we have shown a pronounced immunomodulatory effect of Psd1 on DCs or monocytes as well as on a Caco-2/immune cells co-culture upon a fungal infection [10].

**Figure 4:**
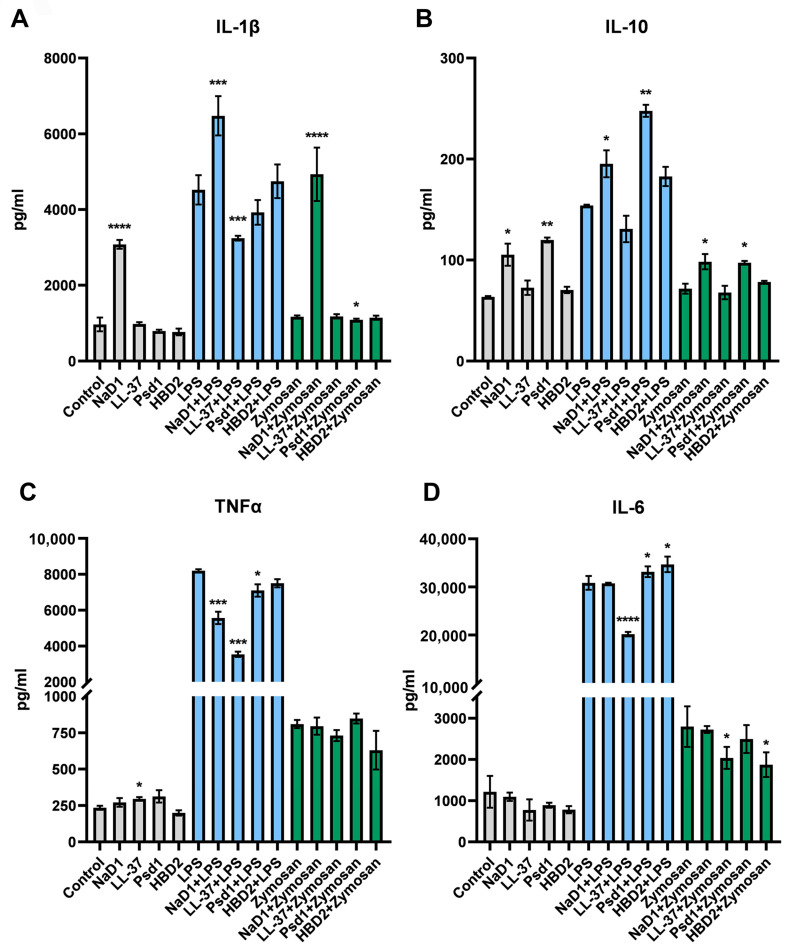
Influence of the tobacco defensin NaD1 and other AMPs at the concentration of 2 µM on production of pro- (**A**–**D**) and anti-inflammatory (**B**) cytokines either unstimulated or stimulated by LPS or by zymosan THP-1-derived macrophages. Error bars represent a standard deviation (±SD) between two biological and two technical replications. Significance levels are * *p* ≤ 0.05, ** *p* < 0.01, *** *p* < 0.001, **** *p* < 0.0001. The significance of difference in cytokine production was calculated by comparing: unstimulated cells (control) with stimulated by AMPs cells (grey bars); stimulated by LPS (blue bars) or zymosan (green bars) cells alone or in the presence of AMPs. Release of the cytokines in control and experimental samples was compared with unpaired two-sample *t*-test.

To perform this analysis, cytokines IL-1β, IL-6, TNF-α and IL-10 were chosen. IL-1β, TNF-α and IL-6 are the major pro-inflammatory cytokines, playing a key role in inflammation, whereas IL-10 is an anti-inflammatory cytokine that suppresses excessive immune responses and antigen-presentation capacity [30]. THP-1-derived macrophages were pre-treated with antimicrobial peptides at concentrations of 2 or 0.2 µM for 2 h followed by stimulation with LPS and zymosan for an additional 24 h. LPS induced the secretion of all cytokines tested, but the most pronounced effect was observed in the case of IL-6 (*p* < 0.0001) and TNF-α (*p* < 0.0001). Zymosan had the same but less pronounced pro-inflammatory effect, and a statistically significant increase in cytokine production was observed only for IL-6 (*p* = 0.0023) and TNF-α (*p* = 0.0015) (Figure 4A–D).

At a concentration of 2 µM, AMPs by themselves had the following effects on THP-1-derived macrophages. Only NaD1 significantly increased IL-1β level (*p* < 0.0001). Plant defensins NaD1 (*p* = 0.0329) and Psd1 (*p* = 0.001) induced the production of IL-10. Among all tested AMPs, only LL-37 slightly increased TNF-α secretion (*p* = 0.0454). All AMPs did not influence IL-6 production (Figure 4A–D).

Along with that, all AMPs in different ways influenced the LPS-induced pro-inflammatory response of THP-1-derived macrophages. As expected, LL-37 inhibited the LPS-induced production of all cytokines tested, although for IL-10 this effect was insignificant. Among defensins, only NaD1 increased the LPS-induced production of IL-1β (*p* = 0.0009). Plant defensins NaD1 and Psd1 increased the LPS-induced production of IL-10, but reduced the LPS-induced secretion of TNF-α. On the contrary, cell pre-incubation with Psd1 (*p* = 0.0436) and HBD2 (*p* = 0.0121), but not with NaD1, increased the LPS-stimulated production of IL-6 (Figure 4A–D).

AMPs also affected zymosan-induced inflammation. LL-37 slightly decreased the zymosan-induced production of TNF-α and IL-6, although only for IL-6 this effect was significant (*p* = 0.0349). NaD1 in the presence of zymosan induced the production of IL-1β (*p* <0.0001) more strongly than without this PAMP. At the same time, Psd1 slightly reduced the production of IL-1β (*p* = 0.0121) in the presence of zymosan. NaD1 (*p* = 0.0116) and Psd1 (*p* = 0.0202) increased the production of IL-10 in the presence of zymosan, but these effects were less pronounced than in the case of individual AMPs. All of the three defensins did not influence the TNF-α production. HBD2 caused a slight decrease in the zymosan-induced production of IL-6 (*p* = 0.0184) (Figure 4A–D).

At the concentration of 0.2 µM, AMPs by themselves or in the presence of PAMPs had approximately the same action, but the effects were generally less pronounced (Supplementary Materials, Figure S3).

LL-37, also known as hCAP18, has been widely shown to exhibit different effects on various types of human cells. In particular, LL-37 suppresses the LPS-induced cell inflammatory response via the direct binding of LPS [31]. As shown, LL-37 interacts not only with LPS but also with polysaccharides from fungal cell walls, such as mannan, chitin and glucan [32]. In our experiments, we observed similar effects of LL-37 on the LPS- and zymosan-induced production of TNF-α and IL-6, probably due to the ability of this peptide to also bind the fungal PAMP zymosan.

As is known, HBD2 modulated various immune processes. It has been reported that pre-stimulation of primary human macrophages or THP-1 cells with HBD2 and its subsequent removal from the culture medium resulted in the enhanced production of pro-inflammatory cytokines and chemokines induced following cell stimulation by LPS or zymosan, apparently through activation of the P2X7 receptor [19]. At the same time, co-treatment with HBD2 at different concentrations mitigated the release of TNF-α and IL-1β by LPS-stimulated PBMCs [33]. In our case, pre-treatment of THP-1-derived macrophages with HBD2 followed by the addition of LPS or zymosan had no significant effect. At the same time, HBD2 increased or, conversely, decreased PAMP-induced IL-6 production in the case of LPS or zymosan, respectively.

As mentioned above, plant defensins also exhibit immunomodulatory effects. In particular, the peptide solyC, corresponding to the γ-motif of the tomato defensin family, affected THP-1 cells stimulated with LPS but had no effect on unstimulated cells. Co-stimulation of THP-1 cells with this peptide and LPS resulted in a decrease of the LPS-induced production of the pro-inflammatory cytokines TNF-α and IFN-γ [9]. According to our data, the plant defensins Psd1 and NaD1 had different effects on unstimulated THP-1-derived macrophages and those stimulated by LPS and zymosan. Both peptides induced the synthesis of the anti-inflammatory cytokine IL-10 by THP-1-derived macrophages, but only NaD1 increased the IL-1β level. Both plant defensins increased IL-10 production and decreased the TNF-α level under LPS-induced inflammation, but NaD1 also significantly increased the secretion of IL-1β and Psd1 slightly increased the IL-6 level. Both plant defensins increased IL-10 production under zymosan-induced inflammation, but only NaD1 also significantly increased the secretion of IL-1β.

As is known, a number of AMPs exhibit immunomodulatory properties and trigger both anti-inflammatory and pro-inflammatory immune responses via distinct mechanisms which depend on biological context [34]. Our data also demonstrated that NaD1 had a pleiotropic action on resting and pro-inflammatory THP-1-derived macrophages, affecting the production of both pro- and anti-inflammatory cytokines. A comparison of the effects of the plant defensins NaD1 and Psd1 revealed that different representatives of plant defensins could have various effects on unstimulated and PAMP-induced pro-inflammatory THP-1-derived macrophages.

Key pro-inflammatory cytokines (IL-1, IL-6 and TNF-α) during infection stimulate the production of acute-phase proteins and attract inflammatory cells. At the same time, an elevated level of IL-6 was found to be associated with the highest risk of death in patients with sepsis [35]. We did not observe any statistically significant changes induced by NaD1 in the production of IL-6. Any immunosuppression or anti-inflammatory signals during the acute-phase response can be responsible for late death in patients with sepsis. For instance, the increase of IL-10 during sepsis was found to be the main predictor of severity and fatal outcome [35]. NaD1 induced an elevation in anti-inflammatory IL-10 production by M0 and M1 macrophages; however, this elevation was mild (from 150 to 180 pg/mL), while the increase in pro-inflammatory IL-1β was much more profound (from 4500 to 6500 pg/mL). Taken together, we demonstrated in this study that the tobacco defensin NaD1 can impact macrophages by inducing changes in the production level of the key cytokines involved in the acute-phase response.

### 2.5. Possible LPS- and Zymosan-Binding Capacity of the Tobacco Defensin NaD1

As mentioned above, LL-37 (pI 10.61) binds to bacterial LPS and polysaccharides from fungal cell walls, including β-glucan, and electrostatic interactions have been shown to play an important role in peptide–PAMP interaction [31,32]. At the same time, NaD1 is cationic peptide with pI 9.08 that could potentially bind to the negatively charged PAMPs. The ability of the tobacco defensin NaD1 to bind β-glucan and chitin has been shown previously [6,36]. To the best of our knowledge, nothing is known about the ability of NaD1 to interact with LPS. In order to test this and estimate the possible influence of PAMP-binding on immunomodulatory effects, in particular, a decrease in the LPS-induced production of TNF-α, we examined the antimicrobial activity of NaD1 in the presence of LPS or zymosan. LL-37 was used for the comparison in these experiments.

The tobacco defensin NaD1 is known to have a pronounced antifungal activity [6,14,15,16]. We tested the activity of this peptide against *Candida albicans* ATCC 18804. Taking into consideration a significant decrease in the activity of NaD1 in the presence of sodium chloride at physiological concentrations, a low-salt media such as Sabouraud broth was used. NaD1 effectively inhibited the growth of *C. albicans* at MIC 6.25 μM. LL-37 also exhibited anticandidal activity at MIC 12.5 μM under the test conditions. The next step was to test the influence of LPS and zymosan on the antifungal activity of AMPs.

The antifungal activity of LL-37 was reduced in the presence of both LPS and zymosan (Table 1). Both PAMPs at concentrations of 40 μg/mL and higher doubled the MIC of LL-37 against *C. albicans.* Higher concentrations of LL-37 were also required for the fungicidal effect on yeast-like cells in the presence of LPS or zymosan at concentrations of 100 μg/mL. At the same time, the presence of LPS or zymosan had a slight effect on the anticandidal activity of NaD1. An increase of the MIC of NaD1 was observed only in the presence of LPS at the concentration of 100 μg/mL. The fungicidal effect of NaD1 decreased in the presence of zymosan or LPS. Plating the contents of the wells with NaD1 in MIC concentrations showed a larger number of colonies of *C. albicans* cells in the case of a higher concentration of LPS or zymosan (Supplementary Materials, Figures S4 and S5). A doubling of the MFC, but not MIC, of NaD1 was found in the presence of zymosan at concentrations of 40 μg/mL and higher (Table 1). Thus, we assumed that NaD1 could potentially bind LPS, but to a lesser extent than LL-37. However, unlike LL-37, the immunomodulatory effects of NaD1 most likely did not relate to its ability to bind the PAMPs used.

### 2.6. Limitations

In this study, we showed that the tobacco defensin NaD1 exhibits different immunomodulatory effects on various immune cells, including monocyte-derived dendritic cells, primary blood monocytes and THP-1-derived macrophages. However, a plenty of cells other than the immune cells used are involved in various immune responses, including in the acute-phase response during infection. This is why the overall immunomodulatory action of NaD1 hard to be predicted ex vivo and need to be further investigated in inflammatory mouse models. It is also worth noting that the safety of NaD1 should be tested due to the cytotoxic activity of the peptide, which it exhibits in high concentrations.

## 3. Materials and Methods

### 3.1. Materials

*Candida albicans* ATCC 18804 was kept at −70 °C in 10% non-fat milk with 10% glycerol. Synthetic melittin (>98% pure) was provided by Dr. Sergey V. Sychev in M.M. Shemyakin and Yu.A. Ovchinnikov Institute of Bioorganic Chemistry of the Russian Academy of Sciences (Moscow, Russia).

### 3.2. Recombinant Production of Antimicrobial Peptides

The pea defensin Psd1 (UNIPROT P81929) was obtained as described previously [9]. Recombinant tobacco defensin NaD1 (UNIPROT Q8GTM0), human cathelicidin LL-37 (UNIPROT P49913) and human β-defensin 2 (HBD2, UNIPROT O15263) were obtained by heterologous expression in E. coli cells (Supplementary Materials, Figure S2). DNA fragments encoding AMPs were synthesized using PCR with overlapping primers and inserted into the expression plasmid vector pET-His8-TrxL (Supporting Materials, Figure S6 and Figure S7 and Table S2). Correct plasmid assembly was verified by DNA sequencing performed in two directions. Heterologous expression was carried out in E. coli BL-21 (DE3) cells transformed with plasmid constructs pET-His8-TrxL-NaD1/LL-37/HBD2 using 0.2 mM isopropyl β-D-1-thiogalactopyranoside (IPTG) as an inductor. Recombinant peptides were purified from clarified cell lysates using metal chelate chromatography, cyanogen bromide cleavage of the fusion proteins and two-stage reversed-phase high performance liquid chromatography (RP-HPLC). Homogeneity and the identity of the recombinant peptide samples were confirmed by MALDI mass spectrometry and CD spectroscopy (Supplementary Materials, Figures S8, S9 and Table S3).

### 3.3. Human Cell Lines and Cultures

Peripheral blood mononuclear cells (PBMCs) from healthy donors were purchased from American Type Culture Collection (ATCC PCS-800-011). Primary monocytes were isolated from PBMCs by adherence to the plastic surface [37]. For that, PBMCs were thawed, resuspended in RPMI-1640 culture medium (Corning, Corning, NY, USA) without serum at a concentration of 2 × 10^6^ cells/mL, seeded into the wells of a 24-well plate and placed in a humidified CO_2_-intubator (CellXpert C170i, Eppendorf, Hamburg, Germany) for 1 h. After that, non-adherent cells were removed from the wells and attached monocytes were thoroughly washed with PBS and cultured in a CO_2_-intubator in complete RPMI-1640 containing 10% human serum (HS Type AB, Capricorn Scientific, Ebsdorfergrund, Germany) and 1×antibiotic-antimycotic (1×AA) solution (Sigma, St. Louis, MO, USA).

Immature monocyte-derived dendritic cells (moDC) were obtained from primary monocytes by a differentiation protocol involving a cytokine cocktail with IL-4 and GM-CSF [38]. For this, the monocytes were cultured in complete RPMI-1640 containing 10% HS, 1×AA solution, 500 U/mL rhIL-4 (Sci-Store, Moscow, Russia) and 800 U/mL rhGM-CSF (Sci-Store) for 3 days. Then, the cells were re-fed with the fresh medium with rhIL-4 and rhGM-CSF and cultured for 4 more days.

THP-1 (ATCC TIB-202) cells were thawed and cultured in complete RPMI-1640, containing 10% FBS (Capricorn Scientific) and 1×AA solution in CO_2_-intubator. Monocytic THP-1 cells were differentiated into resting M0 macrophages by stimulation for 24 h with 100 ng/mL phorbol 12-myristate 13-acetate (PMA) in complete RPMI-1640. Then, the attached macrophage-like cells were washed out with culture medium without serum and incubated for another 48 h in PMA-free complete culture medium with 10% HS and 1×AA solution.

Caco-2 (ATCC HTB-37) cells were thawed and cultured in complete DMEM/F-12 culture medium (Corning), supplemented with 10% FBS in a humidified CO_2_-intubator. After the culture was subcultivated three times, the cells were seeded on cell inserts (PET, 0.4 μm, 0.6 cm^2^ surface area, SPL Life Sciences, Pocheon-si, Republic of Korea) at a density of 2.5 × 10^5^ cells/cm^2^. Cells were grown for 21 days with re-feeding with the fresh medium every 2–3 days. To evaluate integrity of Caco-2 monolayer, a transepithelial electrical resistance (TEER) was measured by Millicell ERS-2 Voltohmmeter (Merck-Millipore, Burlington, MA, USA). Only inserts with TEER > 400 Ω cm^2^ were used in the transport assay.

*C. albicans* ATCC 18804 cells in stock were inoculated onto modified YPD (yeast extract 5 g/L, peptone 10 g/L, glucose 10 g/L) agar plates and incubated for 24 h at 37 °C.

### 3.4. Cytotoxicity Assay

The cytotoxic effects of the tobacco defensin NaD1 towards PBMCs or Caco-2 cells were investigated in 96-well plates using the resazurin method as previously described [10]. In brief, 4 × 10^5^ per well Caco-2 cells not in a monolayer as well as Caco-2 cells in a monolayer in DMEM/F12 (1:1) medium with 10% FBS or 2 × 10^6^ PBMCs per well in RPMI-1640 also with 10% FBS were incubated with serial dilutions of NaD1 at final concentrations from 0.39 to 50 μM or from 2.1 to 180 μM for 24 h. After that, resazurin (Sigma, St. Louis, MO, USA) was added at a final concentration of 70 µM and the plates were incubated overnight (16–18 h). Untreated cells and cells treated by non-ionogenic detergent Triton X-100 were used as negative and positive controls, respectively. The membrane-active peptide melittin from honeybee venom was used for comparison. The cell viability was estimated by resorufin fluorescence registered at 595 hm via the following equation: cell viability (%) = (F_sample_/F_control_) × 100%. Experiment was carried out twice in duplicate.

### 3.5. Labeling of the Tobacco Defensin NaD1 with FITC

The labeling of NaD1 with FITC was performed as previously reported in [21].

### 3.6. Caco-2 Permeability Assay

The permeability assay was conducted in transfer buffer (HBSS solution, containing 1 mM CaCl_2_, 1 mM MgCl_2_, 10 mM D(+)glucose, pH 7.4). For the measuring transport of NaD1 in the absorptive direction (from the apical to basolateral chamber, A→B), 0.7 mL of the transport buffer was placed in the basolateral chamber and 0.4 mL of 5 µM FITC-labeled NaD1 in the transport buffer was placed in the apical chamber. For the measuring transport of NaD1 in the secretory direction (from the basolateral to apical chamber, B→A), 0.7 mL of 5 µM FITC-labeled NaD1 was placed in the basolateral chamber and 0.4 mL of the transport buffer was placed in the apical chamber. The permeability assay through the Caco-2 polarized monolayer was conducted for 90 min in 6 independent inserts for the absorptive direction and 4 independent inserts for the secretory direction. The Caco-2 permeability assay was performed twice on two different days.

The apparent permeability coefficients (Papp) were calculated for each insert according to the following equation: Papp = dQ/dt × (1/(A × C0)), where dQ/dt is an amount of product present in the basolateral or apical chamber as a function of time (nM/s), A is an area of the insert (in cm^2^) and C0 is an initial concentration of NaD1 in the apical or basolateral chamber (nM/mL). In order to verify the cell monolayer, the apparent permeability of a paracellular marker, Lucifer Yellow (Sigma), was estimated.

### 3.7. Stimulation of Human Cell Cultures with NaD1

For the study of the immunomodulatory properties of NaD1, monocytes and immature dendritic cells (moDC) were seeded into the wells of a 24-well plate at densities of 4 × 10^5^ and 1.3 × 10^5^ cells per well, respectively, in complete RPMI-1640 with 10% HS and AA solution. Then, 24 h later, the culture medium was replaced by a fresh one either with 2 μM NaD1 for stimulation of the cells or without if for the control wells. The cultures were kept in a humidified CO_2_-intubator for 24 h and then the samples of the culture media were taken and frozen.

THP-1-derived macrophages were seeded into the wells of a 24-well plate at a density of 2.7 × 10^5^ cells/well in complete RPMI-1640 with 10% HS and 1×AA solution. Then, 24 h later, the culture medium was replaced by the fresh complete RPMI-1640 containing lipopolysaccharide from *Escherichia coli* 0111:B4 (LPS, Sigma, St. Louis, MO, USA) or zymosan from the cell wall of *Saccharomyces cerevisiae* (Serva, Heidelberg, Germany) at concentrations of 1 or 10 μg/mL, respectively; antimicrobial peptides NaD1, Psd1, LL-37 or HBD2 at concentrations of 2 or 0.2 µM; and combinations of antimicrobial peptides with LPS or zymosan at the same concentrations. Insoluble zymosan was resuspended in sterile water and sonicated twice for 30 s; after that, the stock solution was heated to 80 °C for 20 min. Peptides were added 2 h before stimulation with LPS and zymosan. After that, PAMPs solutions were added and the cells were cultured for 24 h. Complete RPMI-1640 only was used in the control wells. Culture supernatants were collected and stored at −70 °C prior to the cytokines’ assessment.

### 3.8. Multiplex Assessment of Cytokine Production by Monocytes and Immature Dendritic Cells upon Stimulation with NaD1

The absolute levels of 48 cytokines, chemokines and growth factors were determined at a protein level by multiplex xMAP technology (Luminex, Austin, TX, USA). For this, a MILLIPLEX Human Panel A kit (HCYTA-60K-PX48, Merck, Darmstadt, Germany) was used and the following 48 analytes were evaluated in two biological replications: sCD40L, EGF, CCL11/Eotaxin-1, FGF-basic/FGF-2, Flt-3 ligand, CX3CL1/Fractalkine, G-CSF, GM-CSF, GROα, IFNα2, IFN-γ, IL-1α, IL-1β, IL-1RA, IL-2, IL-3, IL-4, IL-5, IL-6, IL-7, CXCL8/IL-8, IL-9, IL-10, IL-12(p40), IL-12(p70), IL-13, IL-15, IL-17A/CTLA8, IL-17E/IL-25, IL-17F, IL-18, IL-22, IL-27, CXCL10/IP-10, CCL2/MCP-1, CCL7/MCP-3, M-CSF, CCL22/MDC, CXCL9/MIG, CCL3/MIP-1α, CCL4/MIP-1β, PDGF-AA, PDGF-AB/BB, CCL5/RANTES, TGF-α, TNF-α, TNF-β and VEGF-A. The fluorescent data were obtained on a MAGPIX system (Merck) operated with xPONENT 4.2 software (Merck). Final analysis was performed in MILLIPLEX Analyst v5.1 software (Merck).

### 3.9. ELISA Assay of Cytokine Production by THP-1-Derived Macrophages

The production of pro- and anti-inflammatory cytokines IL-1β, IL-6, TNF-α and IL-10 by stimulated and non-stimulated THP-1-derived macrophages were estimated by using ELISA kits (Vector-Best, Koltsovo, Russia). Briefly, 96-well plates with adsorbed monoclonal antibodies to the corresponding cytokine were used. Undiluted or diluted 5, 20 or 80 times in the case of IL-10, TNF-α and IL-1β or IL-6, respectively, culture supernatants from the wells of 24-well plates were taken for analysis. Biotinylated polyclonal antibodies to cytokines, streptavidin conjugated to HRP and TMB substrate were further used for the detection of immune complexes. A panel of calibration solutions containing different concentrations of cytokines was used in each experiment. Experiments were performed using two biological and two technical repeats. The release of the cytokines in the control and experimental samples was compared with an unpaired two-sample *t*-test using GraphPad Prism v.8.0.1 (GraphPad Software, Inc., San Diego, CA, USA). The *p* values ≤ 0.05 were considered significant.

### 3.10. Antifungal Activity of NaD1 and LL-37

The antifungal assay was performed by the microdilution method using 96-well microplates as described [11]. Briefly, *C. albicans* ATTC 18804 cells in stock were inoculated onto Sabouraud agar plates with 2% glucose and incubated for 24 h at 37 °C. After replating, cells were inoculated in Sabouraud broth and cultured at 37 °C for 2 h. The cell concentration was determined using a LUNA-II cell counter (Logos Biosystems, Anyang-si, Republic of Korea). Yeast cells in Sabouraud broth diluted to concentration of 4 × 10^4^ cells/mL were mixed with equal volumes of serial two-fold dilutions of the peptides in water and the plates were incubated at 30 °C for 24 h. The final peptide concentrations in the wells were 25, 12.5, 6.25, 3.13, 1.56, 0.78 and 0.39 μM. Controls without peptides were also tested. The wells of the microplate were previously blocked with 0.1% BSA. Yeast growth was assessed using an inverted light microscope and also by measuring absorbance at 630 nm. The minimum inhibitory concentrations IC_50_ and MIC were defined as the lowest peptide concentrations inhibiting fungal growth by at least 50 and 100%, respectively. To assess the minimum fungicidal concentrations (MFCs), the entire contents of the plate wells with peptides at MIC concentrations and higher were seeded on Sabouraud agar and incubated at 37 °C for 24 h. The MFCs corresponded to the minimal peptide concentration, in which no colony growth was observed.

To estimate the effects of LPS and zymosan on the antifungal activity of NaD1 and LL-37, these PAMPs were added in culture media at concentrations of 10, 40 and 100 μg/mL [39,40]. All of the experiments were performed twice in triplicate.

## 4. Conclusions

In this work, for the first time, we investigated immunomodulatory properties of the plant defensin NaD1 from tobacco flowers, which has a pronounced antimicrobial activity against pathogenic fungi. Using a monolayer of Caco-2 cells as a model system, we showed that NaD1 has the ability to permeate epithelial barriers. Using the multiplex xMAP assay, we revealed that NaD1 at the concentration of 2 μM exhibited an immunomodulatory effect on immune cells, such as primary monocytes and immature dendritic cells. In contrast to other plant defensins, such as the pea Psd1 and the lentil Lc-def, the action of NaD1 is mainly inhibitory, since the production of a wide range of cytokines/chemokines/growth factors by these cells is reduced in the presence of NaD1. Moreover, using the sandwich ELISA method, we demonstrated that NaD1, also at the concentration of 2 μM, had a pronounced immunomodulatory effect on unstimulated THP-1-derived macrophages and those stimulated by bacterial LPS or fungal zymosan. We showed that NaD1 had a pleiotropic action on resting and LPS- or zymosan-stimulated pro-inflammatory THP-1-derived macrophages, affecting the production of both pro- and anti-inflammatory cytokines. We also noted that the immunomodulatory effects of NaD1 on THP-1-derived macrophages under inflammation in vitro were somewhat different from those of the pea Psd1 and it was unlikely that these effects were due to the ability of tobacco defensin to bind such PAMPs as LPS or zymosan. These data demonstrated a lack of uniformity in the immunomodulatory action of plant defensins and suggested that not only differences in antimicrobial activities, but also various effects on the human immune system could influence the effectiveness of these peptides under infection in vivo. Our results indicate the need for further in vitro and in vivo study of the immunomodulatory effects of these plant AMPs, including a mice model of acute inflammation induced by LPS or other TLR-stimulating agents.

## Figures and Tables

**Table 1:** Effects of LPS and zymosan on antifungal activities of the tobacco defensin NaD1 and the human cathelicidin LL-37 towards *Candida albicans* ATCC 18804.

Test Variant	NaD1, μM			LL-37, μM		
	IC_50_	MIC	MFC	IC_50_	MIC	MFC
Without PAMP	3.12–6.25	6.25	12.5	6.25–12.5	12.5	25
10 μg/mL LPS	3.12–6.25	6.25	12.5	6.25–12.5	12.5	25
40 μg/mL LPS	3.12–6.25	6.25	12.5	6.25–12.5	**25**	25
100 μg/mL LPS	3.12–6.25	**12.5**	12.5	**12.5–25**	**25**	**>25**
10 μg/mL Zymosan	3.12–6.25	6.25	12.5	6.25–12.5	12.5	25
40 μg/mL Zymosan	3.12–6.25	6.25	**25**	**12.5**	**25**	**>25**
100 μg/mL Zymosan	3.12–6.25	6.25	**25**	**12.5–25**	**25**	**>25**

MIC values of the peptides in the presence of PAMPs exceeding those without them are shown in bold.

## Data Availability

Data are contained within the article and Supplementary Materials.

## Supplementary Materials

The following supporting information can be downloaded at: https://www.mdpi.com/article/10.3390/antibiotics13111101/s1, Figure S1: Cytotoxic effects of the tobacco defensin NaD1 at high concentrations towards PBMCs (A) and Caco-2 cells not reached monolayer (B); Figure S2: Comparison of the amino acid sequences of antimicrobial peptides used; Figure S3: Influence of the tobacco defensin NaD1 and other AMPs at concentration of 0.2 µM on production of pro- and anti-inflammatory cytokines by unstimulated or stimulated by LPS or zymosan THP-1-derived macrophages; Figure S4: Effect of LPS on candidacidal action of the tobacco defensin NaD1 and human cathelicidin LL-37; Figure S5: Effect of zymosan on candidacidal action of the tobacco defensin NaD1 and human cathelicidin LL-37; Figure S6: Agarose electrophoresis of amplicons encoding LL-37 (A), NaD1 (C) and HBD2 (E) and assembled plasmid constructs pET-His8-TrxL-LL-37 (B), pET-His8-TrxL-NaD1 (D) and pET-His8-TrxL-HBD2 (F); Figure S7: Schematic representation of the plasmid vectors pET-His8-TrxL-NaD1/LL-37/HBD2; Figure S8: MALDI mass spectra of the recombinant antimicrobial peptides; Figure S9: Circular dichroism spectra of antimicrobial peptides (0.3 mM) in an aqueous solution and in micellar detergents (30 mM); Table S1. Absolute levels of 48 cytokines, chemokines and growth factors assessed by multiplex xMAP technology; Table S2: List of overlapping primers; Table S3: Antimicrobial peptide secondary structure estimation (%) predicted from far-UV CD spectra.
