# Supplementary material for: Immunomodulatory Effects of the Tobacco Defensin NaD1
Source: Antibiotics (Basel). 2024 Nov 19;13(11):1101. doi: 10.3390/antibiotics13111101 (PMC11591356; doi:10.3390/antibiotics13111101)
Supplement: Supplementary file 1 [file antibiotics-13-01101-s001.zip › antibiotics-3277192-supplementary.pdf]

Supplementary Materials for

Immunomodulatory Effects of Tobacco Defensin NaD1

Ekaterina I. Finkina [1,*,†], Ivan V. Bogdanov [1,†], Olga V. Shevchenko [1,2], Serafima I. Fateeva [1,3], Anastasia A. Ignatova [1], Sergey V. Balandin [1] and Tatiana V. Ovchinnikova [1,2,3]

1 M.M. Shemyakin and Yu.A. Ovchinnikov Institute of Bioorganic Chemistry, Russian Academy of Sciences, 117997 Moscow, Russia; ovch@ibch.ru (T.V.O.)
2 Moscow Center for Advanced Studies, 123592 Moscow, Russia
3 Department of Bioorganic Chemistry, Lomonosov Moscow State University, 119991 Moscow, Russia
* Correspondence: finkina@mail.ru; Tel.: +7-495-335-0900
† These authors contributed equally to this work.

Materials and Methods

Assembly of genetic constructs

DNA fragments encoding human peptide cathelicidin LL-37, plant defensin and human β-defensin HBD2 were obtained by de novo synthesis using PCR with overlapping primers (Supplementary materials, Table S2) and Tersus polymerase (Evrogen, Russia). The following thermocycling conditions were used: 95°C – 1 min; then 30 amplification cycles (95°C - 30 s, 55° - 15 s, 72°C - 15 s); then 72°C – 2 min. The quality of the synthesised DNA fragments for LL-37 (163 bp), NaD1 (197 bp) and HBD2 (182 bp) was assessed by electrophoresis in 1.5% agarose gel and ChemiDoc XRS+ gel documentation system (Supplementary materials, Figure S5).

Then DNA fragments were inserted into the expression plasmid vector pET-His8-TrxL, which was pre-treated with BamHI (Thermo Fisher Scientific, Inc., Waltham, MA, USA). The expression plasmid pET-His8-TrxL-HBD2 was constructed by the Gibson assembly method using 2x Gibson Assembly®Master Mix (New England Biolabs, Ipswich, MA, USA) and the expression plasmids pET-His8-TrxL-LL-37/NaD1 were constructed by the ligase-free cloning method using Antarctic Phosphatase (New England Biolabs, Ipswich, MA, USA) (Supplementary materials, Figure S6). Note that pET-His8-TrxL-LL-37/NaD1 was constructed using pET32(a)+ backbone, while pET-His8-TrxL-HBD2 was constructed upon the smaller (lacking lacI gene) pET-20b backbone. This difference was not dictated by the need for more or less strict control of basal expression, but was due solely to the convenience of genetic engineering procedures. Other accessory and regulatory elements were exactly the same in both cases. Then the reaction mixtures were used to transform competent *E. coli* DH10B cells. Right plasmid assemble was verified by DNA sequencing performed in two directions.

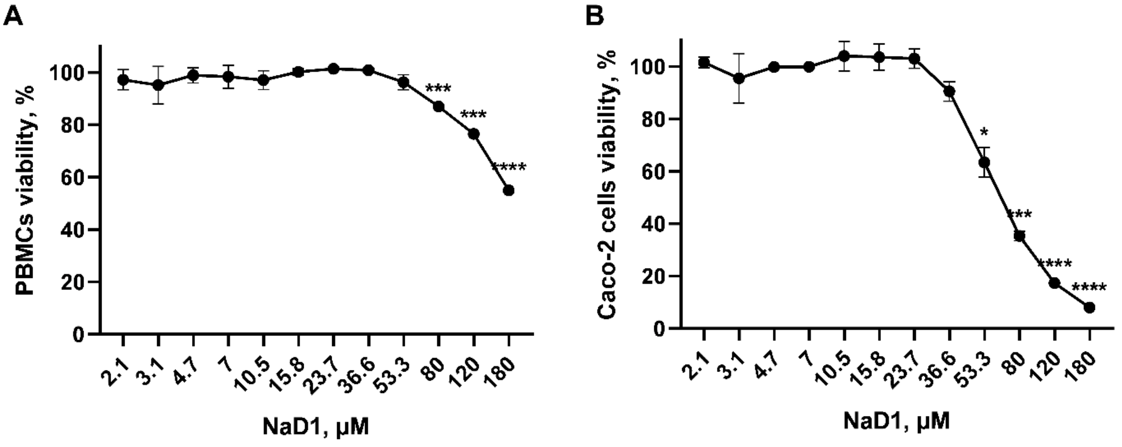

Figure S1. Cytotoxic effects of the tobacco defensin NaD1 at high concentrations towards PBMCs (A) and Caco-2 cells not reached monolayer (B). Error bars represent a standard deviation (±SD) between two biological and two technical replications. Significance levels are: * $p \leq 0.05$, *** $p < 0.001$, **** $p < 0.0001$. The

significance was calculated by comparing untreated cells (control) with treated by NaD1 cells. Viability cells in control and experimental samples was compared with un-paired two-sample *t*-test.

		Mm	pI
**NaD1**	RECKTESNTFPGICITKPP-CRKA-CISEKFTD-GHCSKILRRCLCTKPC	5296.3	9.08
**Psd1**	KTCEHLADTYRGVCFTNAS-CDDH-CKNKAHLISGTCHNWK--CFCTQNC	5200.9	7.73
**Lc-def**	KTCENLSDSFKGPCIPDGN-CNKH-CKEKEHLLSGRCRDDFR-CWCTRNC	5441.2	8.20
**HBD2**	GIGDPVTCLKSGAICHPVFCPRRYKQI-GTCGLPGTKC-CKKP	4328.0	8.99
**LL-37**	LLGDFFRKSKEKIGKEFKRIVQRIKDFLRNLVPRTES	4439.3	10.61

Figure S2. Comparison of the amino acid sequences of antimicrobial peptides used. Cysteine residues are shown in red. Other identical ones for the three plant defensins – tobacco NaD1, pea Psd1 and lentil Lc-def are highlighted in blue.

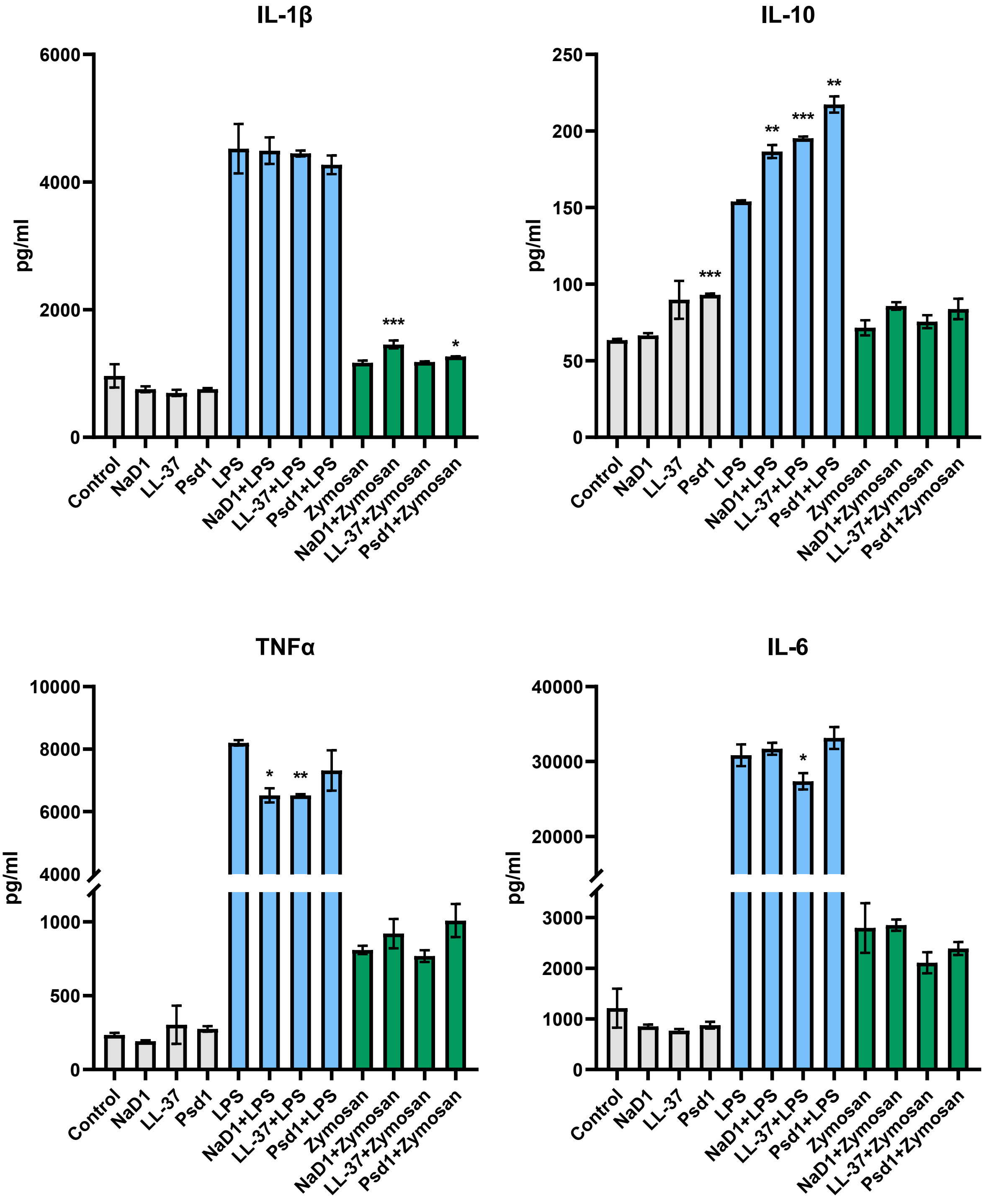

Figure S3. Influence of the tobacco defensin NaD1 and other AMPs at concentration of 0.2 μM on production of pro- and anti-inflammatory cytokines by unstimulated and stimulated by LPS or zymosan THP-1 derived macrophages. Error bars represent a standard deviation (±SD) between two biological and two technical replications. Significance levels are: * $p \leq 0.05$, ** $p < 0.01$, *** $p < 0.001$. The significance of changes in cytokine production was calculated by comparing: unstimulated cells (control) and cells stimulated with AMPs (grey bars); cells stimulated with LPS (blue bars) or zymosan (green bars) alone or in the presence of AMPs. Release of the cytokines in control and experimental samples was compared with unpaired two-sample *t*-test.

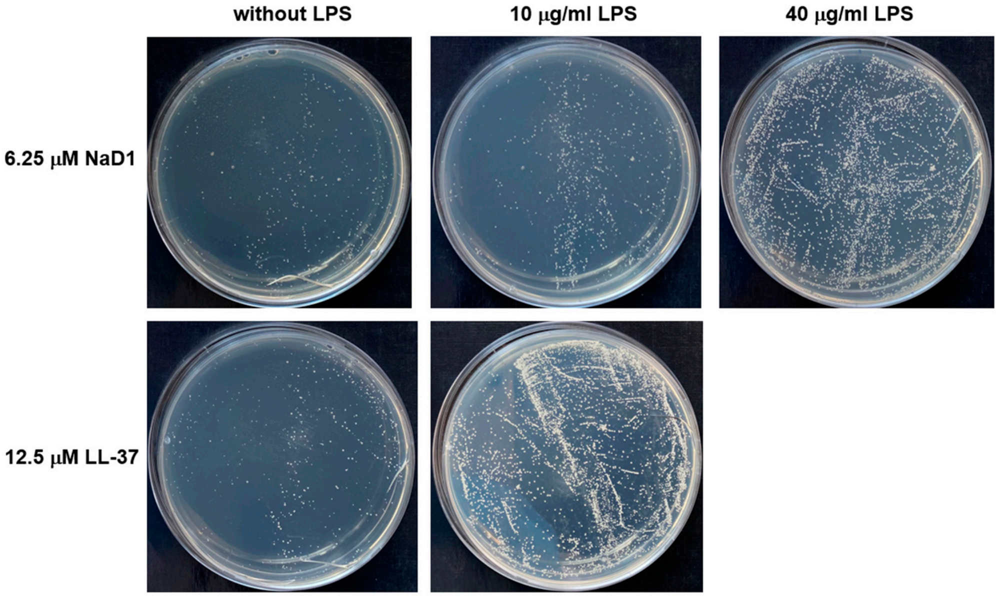

Figure S4. Effect of LPS on candidacidal action of the tobacco defensin NaD1 and human cathelicidin LL-37. The results of plating the contents of the wells with peptides at MIC concentrations in the presence or without LPS onto a solid broth and incubating the plates for 24 hours.

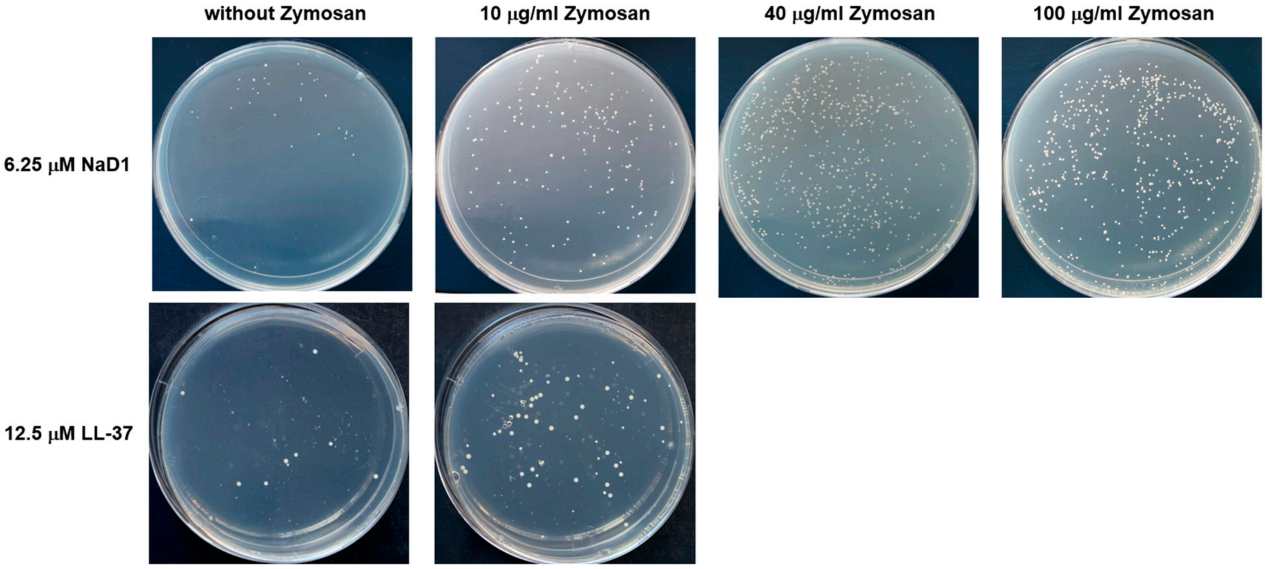

Figure S5. Effect of zymosan on candidacidal action of the tobacco defensin NaD1 and human cathelicidin LL-37. The results of plating the contents of the wells with peptides at MIC concentrations in the presence or without zymosan onto a solid broth and incubating the plates for 24 hours.

Table S2. List of overlapping primers.

LL-37:

№	primer sequences (5′-3′)
Dir1	CCTCGACGCTAACCTGGCCGGATCTATGCTGCTGGGTGATTTCTTCCGTAAAT
Dir2	GCTGGGTGATTTCTTCCGTAAATCTAAAGAGAAGATTGGCAAAGAATTTAAACGTATTGTGCAGC GT
Rev3	CGGTACGCGGCACAAGATTACGCAGAAAATCCTTGATACGCTGCACAATACGTTTAAATTC
Rev4	GGTGCTCGAGAGAATTCGCGGATCCTTAGGACTCGGTACGCGGCACAAGATTA

NaD1:

№	primer sequences (5′-3′)
Dir1	CCTCGACGCTAACCTGGCCGGATCTATGCGTGAATGCAAAACCGAAAGCAATACAT
Dir2	GCAAAACCGAAAGCAATACATTCCCGGGCATTTGCATTACCAAACCACCATGCCGTAAAGCTTG TATCAGTGAGAAAT
Rev3	GCACAGGCAACGACGCAGCAGTTTGCTA CAATGACCA TCGGTAAATTT CTCACTG ATACAAGCTTT
Rev4	GGTGCTCGAGAGAATTCGCGGATCCTTAGCATGGTTTAGTGCACAGGCAACGACGCA

HBD2:

№	primer sequences (5′-3′)
Dir1	CCTCGACGCTAACCTGGCCGGATCTATGGGCATCGGCGACCCGGTTACCTGCCTGAAAAGCGGC GCGATCTGCCACCCTGTATTTGCCCACGGCGTTAT
Rev2	GGTGCTCGAGAGAATTCGCGGATCCTTATTACGGTTTTTTGCAACATTTAGTACCCGGCAGACCG CAGGTACCGATCTGTTTATAACGCCGTGGGCAAAATACAG

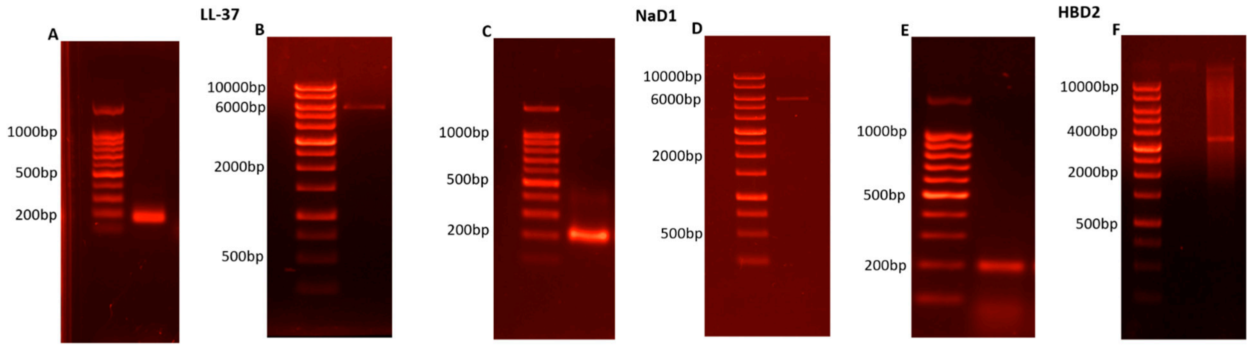

Figure S6. Agarose electrophoresis of amplicons encoding LL-37 (A), NaD1 (C) and HBD2 (E), and assembled plasmid constructs pET-His8-TrxL-LL-37 (B), pET-His8-TrxL-NaD1 (D) and pET-His8-TrxL-HBD2 (F).

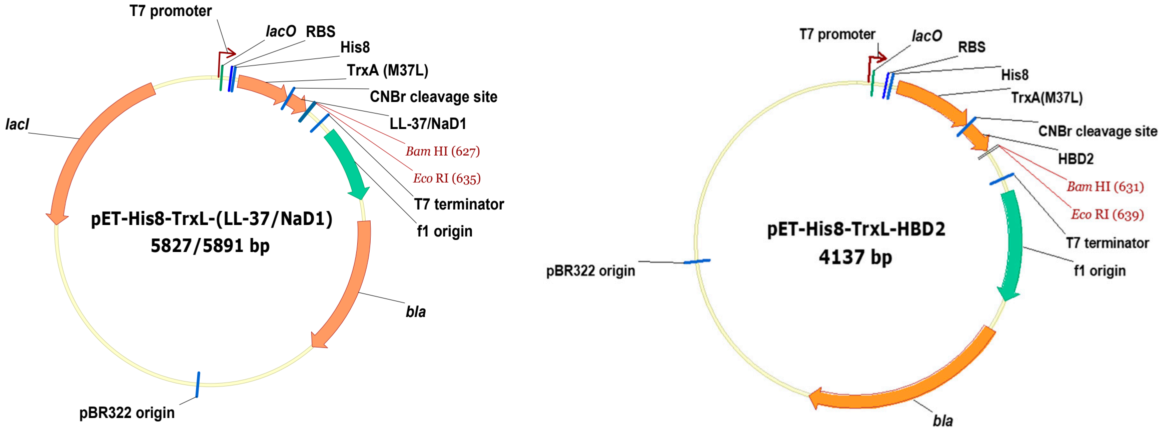

Figure S7. Schematic representation of the plasmid vectors pET-His8-TrxL-NaD1/LL-37/HBD2.

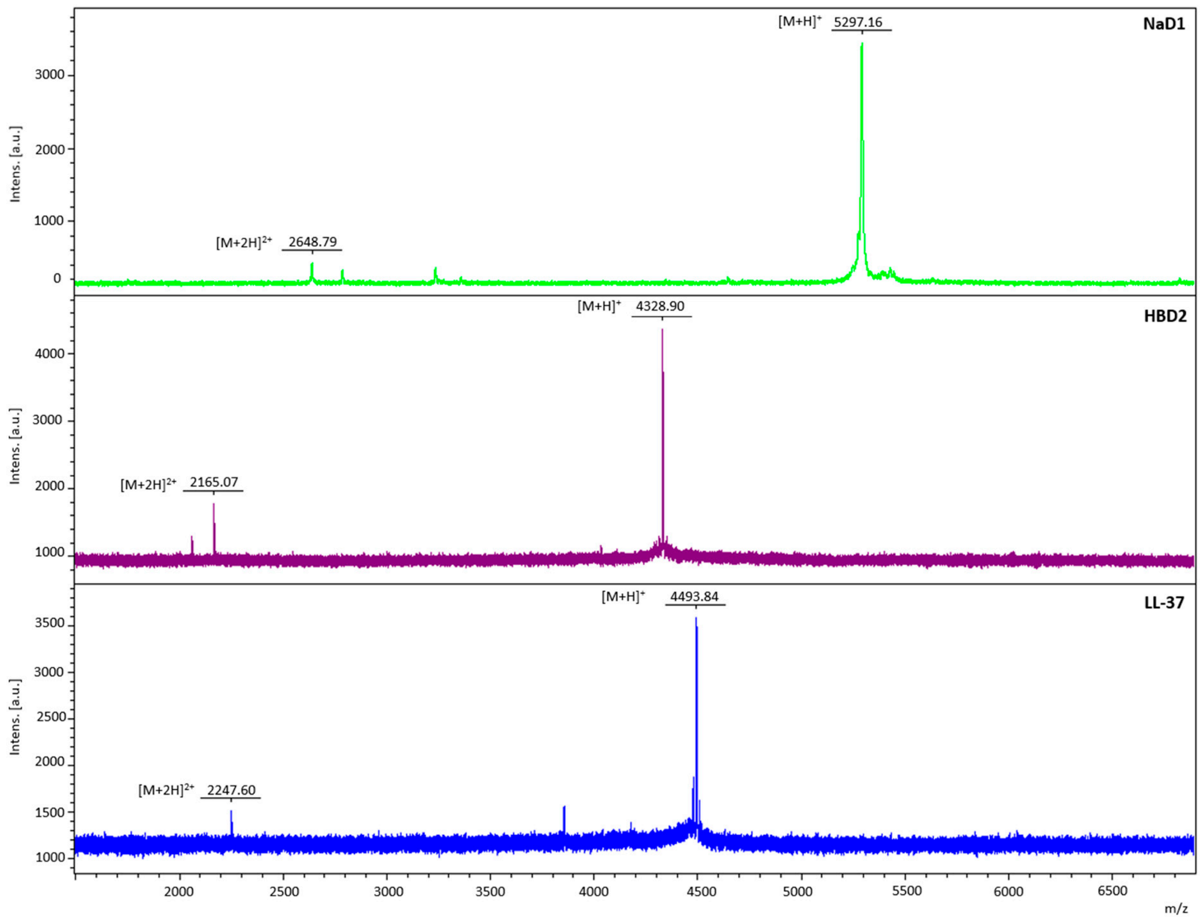

Figure S8. MALDI mass spectra of the recombinant antimicrobial peptides.

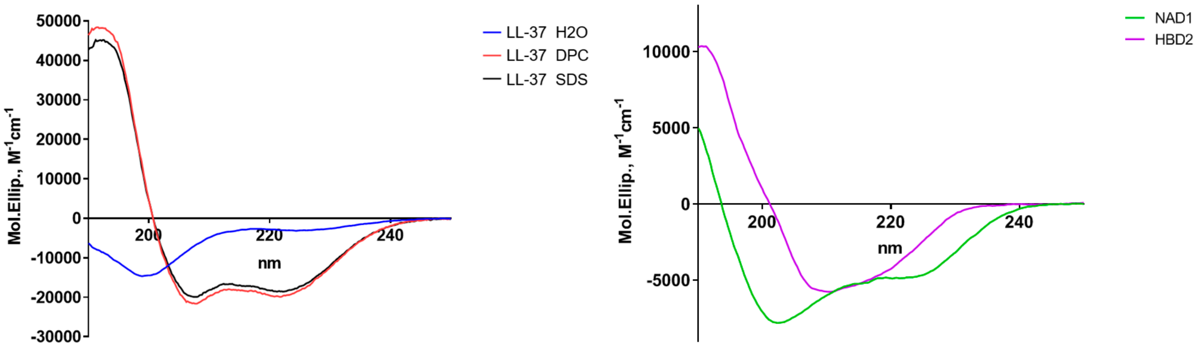

Figure S9. Circular dichroism spectra of antimicrobial peptides (0.3 mM) in an aqueous solution and in micellar detergents (30 mM). Measurements were performed at room temperature using a J-810 spectropolarimeter (JASCO Corp., Japan) in a cuvette with an optical path length of 0.01 cm.

Table S3. Antimicrobial peptide secondary structure estimation (%) predicted from far-UV CD spectra.

Peptide	Condition	α-Helix, %	β-Sheet, %	β-Turn, %	Random, %	NRMSD
NaD1	Aqueous solution	11.4	31.5	21.3	35.9	0.03
HBD2	Aqueous solution	8.2	39.7	20.9	31.3	0.04
LL-37	Aqueous solution	7.9	26.3	24.2	41.6	0.05
	DPC micelles	68.0	4.9	7.5	19.5	0.03
	SDS micelles	64.7	6.5	9.3	19.6	0.03
